# Genome-wide identification, phylogeny, and expression analysis of pectin methylesterases reveal their major role in cotton fiber development

**DOI:** 10.1186/s12864-016-3365-z

**Published:** 2016-12-07

**Authors:** Weijie Li, Haihong Shang, Qun Ge, Changsong Zou, Juan Cai, Daojie Wang, Senmiao Fan, Zhen Zhang, Xiaoying Deng, Yunna Tan, Weiwu Song, Pengtao Li, Palanga Kibalou Koffi, Muhammad Jamshed, Quanwei Lu, Wankui Gong, Junwen Li, Yuzhen Shi, Tingting Chen, Juwu Gong, Aiying Liu, Youlu Yuan

**Affiliations:** 1 State Key Laboratory of Cotton Biology, Key Laboratory of biological and genetic breeding of cotton, Institute of Cotton Research, Chinese Academy of Agricultural Sciences, Anyang, 455000 Henan China; 2 State Key Laboratory of Cotton Biology, Key Laboratory of Plant Stress Biology, College of Life Science, Henan University, Kaifeng, 475004 China

**Keywords:** Cotton, Pectin methylesterases (*PMEs*), Gene family, Gene structure, Phylogeny, Expression patterns

## Abstract

**Background:**

Pectin methylesterase (PME, EC 3.1.1.11) is a hydrolytic enzyme that utilizes pectin as substrates, and plays a significant role in regulating pectin reconstruction thereby regulating plant growth. Pectin is one of the important components of the plant cell wall, which forms the main structural material of cotton fiber. In this research, cotton genome information was used to identify *PMEs*.

**Results:**

We identified 80 (*GaPME01*-*GaPME80*) *PME* genes from diploid *G. arboreum* (A genome), 78 (*GrPME01*-*GrPME78*) *PME* genes from *G. raimondii* (D genome), and 135 (*GhPME001*-*GhPME135*) *PME* genes from tetraploid cotton *G. hirsutum* (AD genome). We further analyzed their gene structure, conserved domain, gene expression, and systematic evolution to lay the foundation for deeper research on the function of *PMEs*. Phylogenetic data indicated that members from the same species demonstrated relatively high sequence identities and genetic similarities. Analysis of gene structures showed that most of the *PMEs* genes had 2–3 exons, with a few having a variable number of exons from 4 to 6. There are nearly no differences in the gene structure of *PMEs* among the three (two diploid and one tetraploid) cotton species. Selective pressure analysis showed that the Ka/Ks value for each of the three cotton species *PME* families was less than one.

**Conclusion:**

Conserved domain analysis showed that *PMEs* members had a relatively conserved C-terminal pectinesterase domain (PME) while the N-terminus was less conserved. Moreover, some of the family members contained a pectin methylesterase inhibitor (PMEI) domain. The Ka/Ks ratios suggested that the duplicated *PMEs* underwent purifying selection after the duplication events. This study provided an important basis for further research on the functions of cotton *PMEs*. Results from qRT-PCR indicated that the expression level of different *PMEs* at various fiber developmental stages was different. Moreover, some of the *PMEs* showed fiber predominant expression in secondary wall thickening indicating tissue-specific expression patterns.

**Electronic supplementary material:**

The online version of this article (doi:10.1186/s12864-016-3365-z) contains supplementary material, which is available to authorized users.

## Background

Cotton (*Gossypium* spp.) is one of the most important natural fiber crops around the world. The improvement of cotton fiber quality is becoming increasingly important and is now a main focal point of cotton research [1, 2]. Pectin is an important component of cotton fiber and pectin metabolism may influence fiber quality. Previous studies showed that *PMEs* play an important role in the process of fiber development by influencing the chemical properties of pectin [1]. Process of cotton fiber cell developing was purposely divided into four relative independent but overlapping stages: fiber initiation, elongation, secondary wall biosynthesis and maturation [3]. Fiber initiation and elongation are critical periods in which the number and lengths of fibers, secondary wall thickening (fiber strength), and other fiber quality traits are determined..The secondary wall thickening in cotton fibers starts 15–19 d after flowering and continues to thicken until 40–50d [4]. The increasing thickness of the fiber secondary wall gradually increases the strength of fibers.

A forward subtractive cDNA library constructed and sequenced from upland cotton (*G. hirsutum*) fibers during the secondary cell wall thickening stage. Computational analysis showed differentially expressed genes that may be involved in cell wall synthesis and modification of biological processes. Among them, several differentially expressed genes which encoded *PMEs* were identified. Thus, in order to elucidate the relationship between *PMEs* and fiber development, we analyzed identification, phylogeny expression of *PMEs* in *G. arboreum*, *G. raimondii* and *G. hirsutum*.

*PMEs* are widely present in plants and some microorganisms that possess a cell wall degradation function. PMEs catalyze the demethylesterification of pectin, which generates carboxyl groups during the release of methanol and hydrogen ions [5]. It plays an important role in cell wall composition modification and degradation if pectin in different development stages of plant, such as fruit maturity [6], pollen development and pollen tube growth [7], cambium cell differentiation, and other plant growth and so on. PMEs have a two-part influence on the cell wall. These produce carboxyl groups and combine with extracellular Ca^2+^ to form a calcium chain bridge between adjacent pectins, thereby hardening the cell wall and slowing cell diffuse growth [8]. And, the reaction of demethylesterification decreases the extracellular pH to increase the hydrolytic enzyme activities of enzymes such as poly-galacturonic acid and several pectin enzyme cleavage enzymes [9]. Pectin is subject to substantial degradation, causes cell wall structure relaxation, and enhances the growth of cell tips [10]. The activity of PMEs is regulated by pectin methylesterase inhibitors (PMEIs) [11] whose active site is the conserved PME domain. All members of PME family consist of a catalytically active zone PME domain; some also harbor a PMEI domain. Some proteins containing only one PMEI domain belong to the PMEI family. Therefore, the predicted proteins can be classified into two categories, type I, containing both PME and PMEI domains, and type II, consisting only a PME domain.

The *PME* belongs to a multigene family which was first described by Richard [12]. There are 66 *PMEs* in Arabidopsis [13], 16 in *Phytophthora sojae* [14], 43 in rice [15], 105 in flax [16], and 81 in *G. raimondii* [1].

Previous reports suggested that *PMEs* may play a part in cell wall development of cotton fibers [1]. At present, studies related to *PME* genes mainly focused on cloning, and functional analysis of single gene [17]; and few analysis had been carried out at the whole genome level [1]. In 2012, the genome of *G. raimondii* was completed [18, 19]. The genome map of cultivated cotton *G. arboreum* was available in 2014 [20]. And next year, the genome map of allotetraploid cultivated cotton (*G. hirsutum* cv TM-l) was completed [21, 22]. The whole genome sequencing of cotton species provides opportunities for comprehensive analysis and comparison of the *PMEs. PMEs*, and its homologous genes were analyzed using bioinformatics analysis on the cotton genome sequence. The results showed that sequence similarities and gene structures were highly conserved. In this study, the gene structure, expression, phylogenetic tree, collinearity of homologous genes and other corresponding analysis were examined systematically by employing the methods of bioinformatics. The results of this study will provide novel insights into research of synthesis mechanism of cotton fiber cell wall.

## Results

### Identification of cotton *PMEs*

**Fig. 1:**
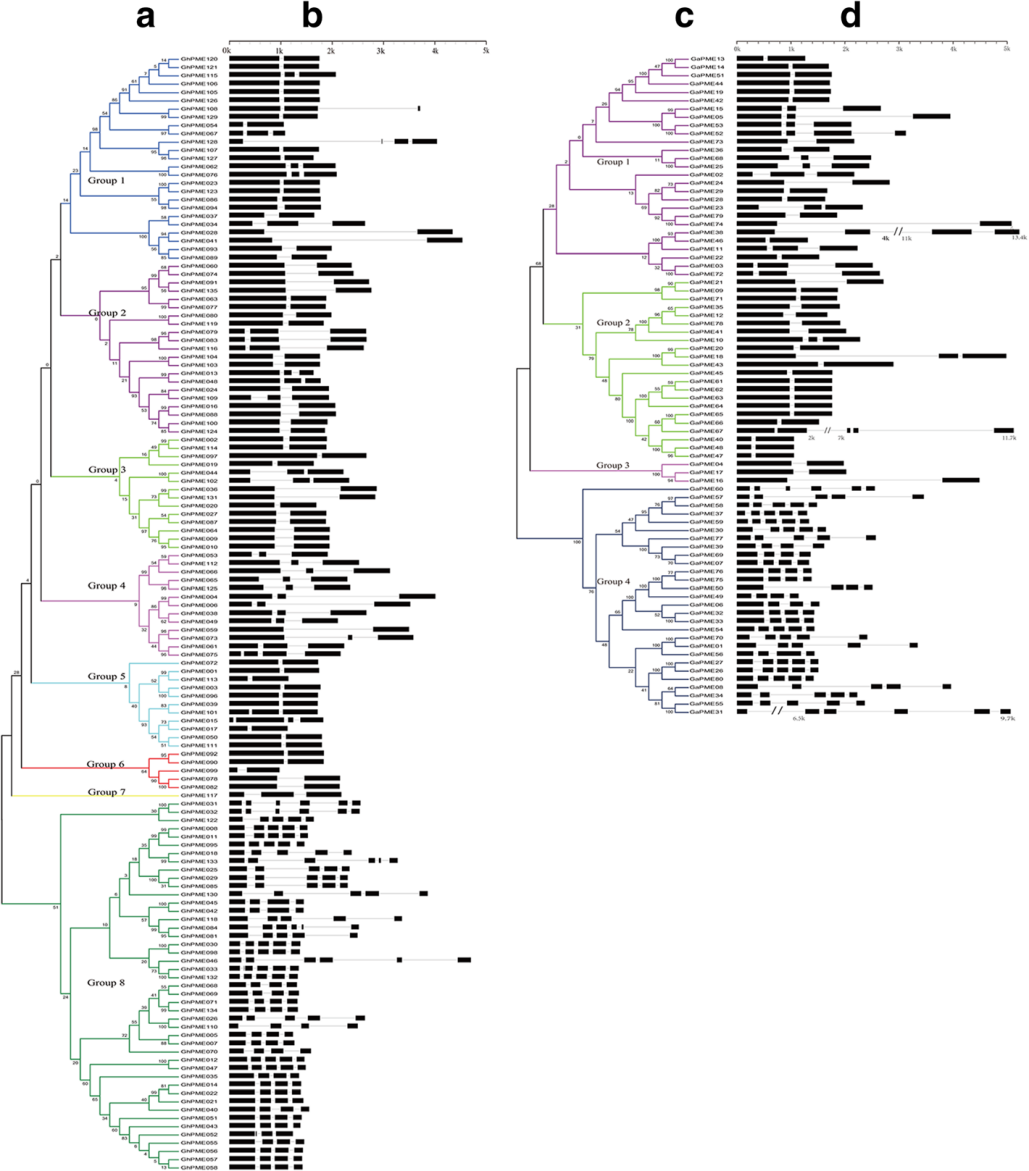
Phylogenetic relationship and gene structure of the *G. arboreum and G. hirsutum PMEs*. **a** A phylogenetic tree was constructed using MEGA 5.1 with the neighbor-joining (NJ) method with 1000 bootstrap replicates based on a multiple alignment of 135 amino acid sequences of PMEs from *G. hirsutum*. The eight major subfamilies are numbered I to VIII. **b** Exon/intron structure of PMEs from *G. hirsutum*. Exons and introns are represented by boxes and black lines, respectively. **c** A phylogenetic tree was constructed with MEGA 5.1 using the neighbor-joining (NJ) method with 1000 bootstrap replicates based on a multiple alignment of 80 amino acid sequences of *PMEs* from *G. arboreum*. The four major subfamilies are numbered I to IV. **d** Exon/intron structure of *PMEs* from *G. arboreum*. Exons and introns are represented by boxes and black lines, respectively

From the three cotton genomes (AD, A, and D), we identified 135 full-length putative *G. hirsutum PMEs* (*GhPME001-135*), 80 full-length putative *G. arboretum PMEs* (*GaPME01*-*GaPME80*), and 78 full-length putative *G. raimondii PMEs* (*GrPME01-GrPME78*) (see Additional file 1: Table S1, and Fig. 1a, c). The family members were named according to their location and sequence on the chromosome.

### Gene structure and protein domain of *PMEs* in different species

The length of the *PMEs* between different cotton species was variable mainly due to large differences in the intron length of each gene. The length of the exons in *PMEs* ranged from 1045 bp to 13398 bp in *G. arboreum,* 1045 bp to 6730 bp in *G. raimondii*, and 964 bp to 4695 bp (with a majority between 1500 bp and 2500 bp) in *G. hirsutum* (Additional file 1: Table S1). The number of amino acid (AA) residues in the GaPMEs protein ranged from 301 to 1169, 316 to 1644 in GrPMEs, and 260 to 845 in GhPMEs (Additional file 1: Table S1). Asiatic cotton *PMEs* gene structure analysis results (Fig. 1d) showed that there were differences between different members. The members of the exon number ranged from 2 to 6, and the gene structure analysis showed that the gene structure of the family members was conserved. The gene structure could be mainly divided into three types. Type I has a typical of two exons and two introns; the differences in the first and second exon were highly conserved, but the length of introns was different. There were 37 such *PMEs* (46.25%) distributed in groups one, two, and three. Type II contained three exons, and there were 12 members (15%) in this group. Among the three exons in this group, the first two exons had significantly different length while the length of the third exon was highly conserved. Type III contained four to six exons with a shorter length than type I or II. These results suggested that the gene structures were similar between of *G. hirsutum* (Fig. 1b) and *G. arboreum* (Fig. 1d).

Eighty members of the *PMEs* family in *G. arboreum* had evolutionary tree clustering relations, and could be divided into four families (Fig. 1c). The analysis of the conserved sequence of the PMEs family members and domain analysis showed that all of them contain a PME domain. Most of the family members of PMEs contained both PME and PMEI. Only five proteins GaPME13 and GaPME46 in group 1, and GaPME40, GaPME47, and GaPME48 in group 2 included only the PME domain. Members of a fourth subfamily contained only a PME domain without a PMEI domain, and there was nearly no difference in *G. hirsutum* (see Additional file 1: Table S1).

### Distribution of *PMEs* family members

We found 80 *PMEs* corresponding to the protein-coding genes in the Asiatic cotton database. These 80 genes were distributed on the 13 chromosomes (Additional file 2: Figure S1b), in which the most *PMEs* (11) were located on chromosome 1 and chromosome 10. Ten *PME*s are mapped on chromosome 2, nine on chromosome 9, eight on chromosome 4, three on chromosome 6, and only one was mapped to chromosome 12. Each of the chromosomes 3, 5, 7, and 8 had four genes. Only one gene was not detected on the chromosome and was positioned on the scaffold. Eighty genes showed uneven distribution on the chromosomes. Some genes arised by tandem duplication. Seven genes (*GaPME61*- *GaPME67*) on chromosome 10 were located on the same block, which we named as cluster I. Five such clusters were located on chromosomes 1, 2, 9, 11, and 13; these clusters covered by 22.5% of *PMEs*. We found 135 *PMEs* in the cotton AD genome (Additional file 2: Figure S1a). Of these, all the 26 chromosomes except At_chr12, fourteen genes (10.4%) were located on chromosome Dt_chr9, 10 were mapped on chromosome At_chr9, and some genes appeared via tandem duplication on chromosomes in cotton AD and D genomes. We found 78 *PMEs*, of which were distributed to all the chromosomes except chromosomes 4, 12. Chromosomes 9, 8, 6, 7, 2, 1, had 16, 10, 8, 6, 2, and 3 chromosomes respectively. Chromosome 10 and 11 together had five genes; chromosome 5 and 13 had three while chromosomes 3 and 12 had one gene (Additional file 1: Table S1).

**Fig. 2:**
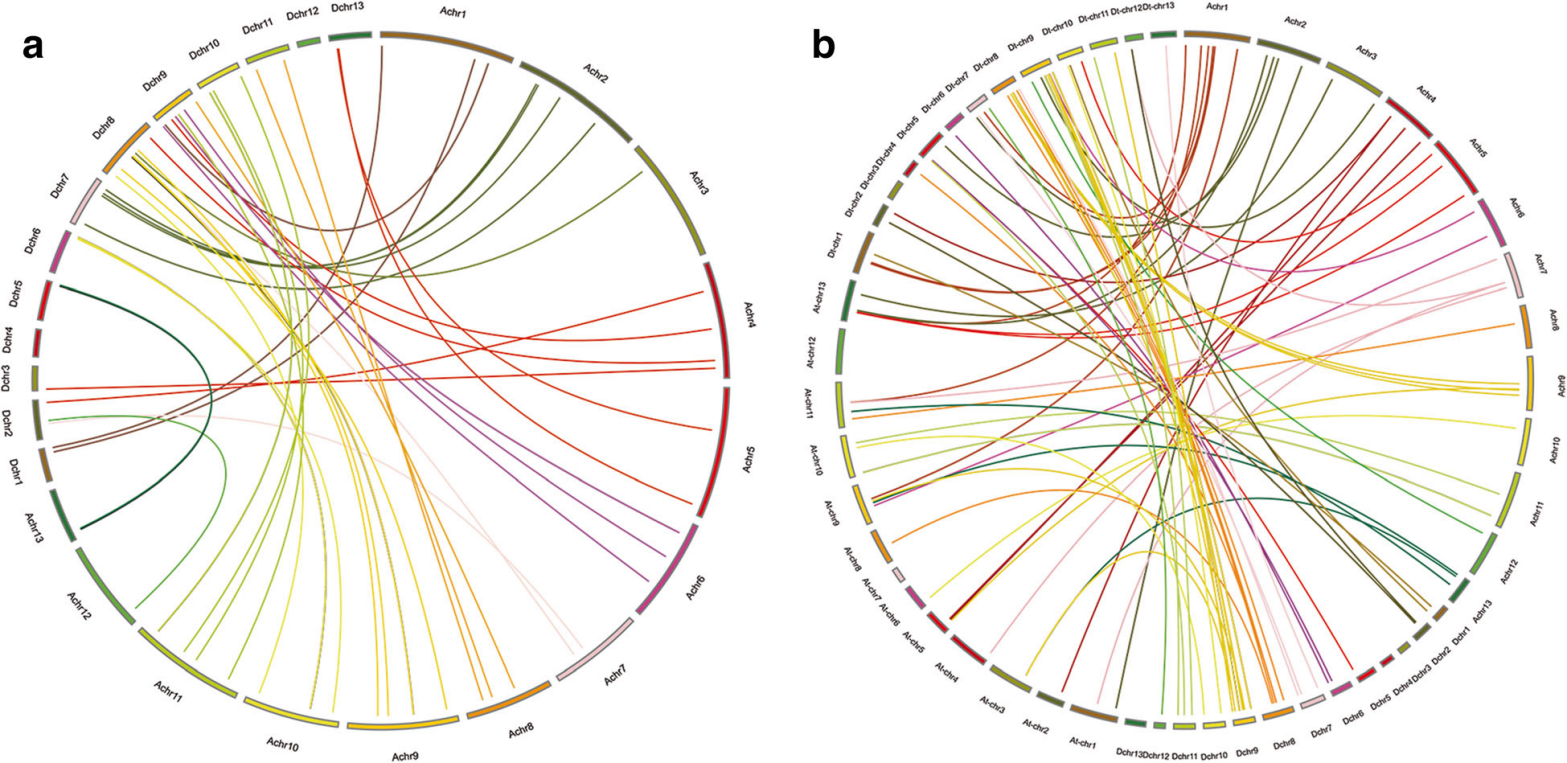
CIRCOS figure of *PME* homologous genes pairs of *G. raimondii and G. arboreum*. **a** CIRCOS figure of *PME* homologous genes pairs of *G. raimondii and G. arboreum*. Lines represent homologous genes that are distributed in syntenic blocks between *G. raimondii* and *G. arboreum* chromosomes. **b** CIRCOS figure of *PME* homologous genes pairs of *G. raimondii and G. hirsutum*, *G. arboreum* and *G. hirsutum*. Lines represent homologous genes that are distributed in syntenic blocks between *G. raimondii, G. arboreum and G. hirsutum* chromosomes

Based on the results of collinearity analysis between *G. raimondii* and *G. arboreum*, 61 homologous gene pairs were distributed in 36 collinearity blocks (Fig. 2a, Additional file 3: Table S2). Among them, one syntonic block contained 19 homologous gene pairs in *G. arboreum* chromosome 10. We identified 57 homologous gene pairs between *G. hirsutum* (Fig. 2b, Additional file 4: Table S3) and *G. arboreum,* and 50 homologous gene pairs between *G. hirsutum* and *G. raimondii* (Fig. 2b, Additional file 4: Table S3). Some genes were not shown in Fig. 2 because they were not positioned on the chromosome (Additional file 4: Table S3).

### Phylogenetic analysis

**Fig. 3:**
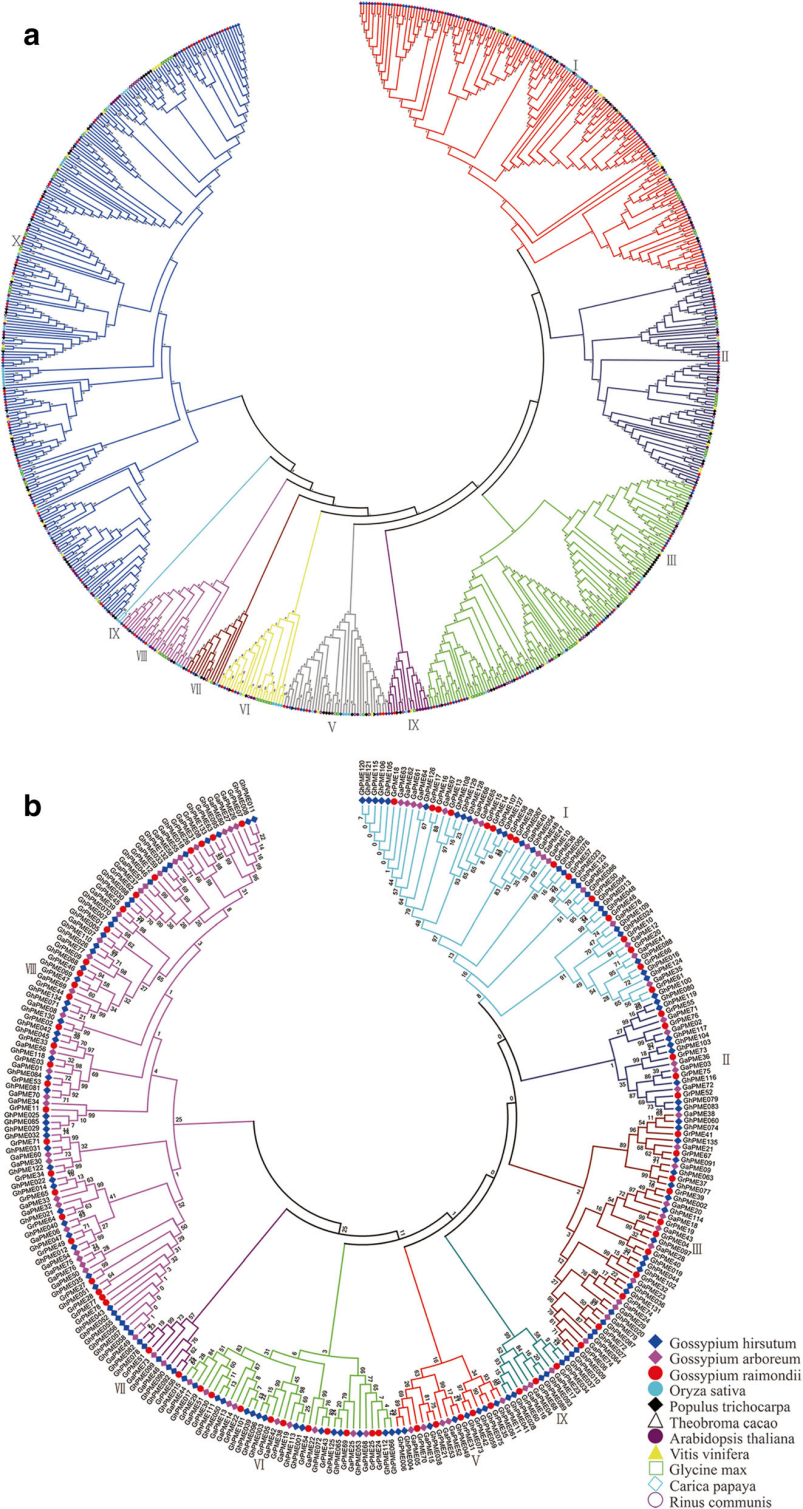
Phylogenetic tree of PMEs. **a** Phylogenetic tree of PMEs from 11 species. The phylogenetic tree is based on a sequence alignment of the C-terminal PME domains of 751 PMEs protein sequences from 11 genomes, *G. hirsutum*, *G. arboreum*, *G. raimondii*, *A. thaliana*, *O. sativa*, *V. vinifera*, *P. trichocarpa*, *G. max*, *T. cacao*, *C. papaya*, and *R. communis*. The PME proteins are grouped into 10 distinct clades (I–X). **b** Phylogenetic tree of PME domain containing proteins from *G. raimondii, G. arboreum, and G. hirsutum.* The phylogenetic tree is based on a sequence alignment of the C-terminal PME domains of 293 PME protein sequences from three genomes, *G. arboreum, G. raimondii, and G. hirsutum.* The tree was generated with MEGA 5.1 using the neighbor-joining method. Bootstrap values from 1000 replicates are indicated at each node. The PME proteins are grouped into 8 distinct clades (I–VIII)

Phylogenetic analysis indicated that PMEs of the same species shared the highest similarities and had relatively close genetic relationships. In order to analyze the evolutionary relationships among the predicted GhPMEs, GaPMEs, and GrPMEs based on amino acid sequence, we aligned cotton amino acid sequences with 458 predicted PMEs from eight sequenced plants such as *A. thaliana*, rice, rice, grape, poplar, soybean, cocoa, papaya, and castor bean. Finally, phylogenetic trees were constructed by using MEGA with the neighbor-joining model. We found that the PMEs family could be divided into 10 subfamilies according to cluster analysis (Fig. 3a). The PMEs had a close genetic relationship within the same species and with cocoa. However, these genes were distant to other species on the evolutionary scale. These data indicated that the *PMEs* might evolve along with different directions for various species. Meanwhile, to examine the evolutionary relationship of *PMEs* in *G. arboreum*, *G. raimondii*, and *G. hirsutum*, the phylogenetic tree was built with 293 *PMEs* in which were divided into eight families (Fig. 3b).

The value of the nonsynonymous substitution rate (Ka) to the synonymous substitution rate (Ks) substitutions (Ka/Ks) can be used as an indicator which could reflect selection pressure of a gene or a gene region during evolution. To infer the influence of selection on the evolution of the three cotton species versus cocoa, we estimated Ka/Ks values for all of them (Additional file 5: Table S4). Our results suggested that all of the three cotton species evolved mainly under the influence of stabilizing selection.

### Transcriptome analysis

**Fig. 4:**
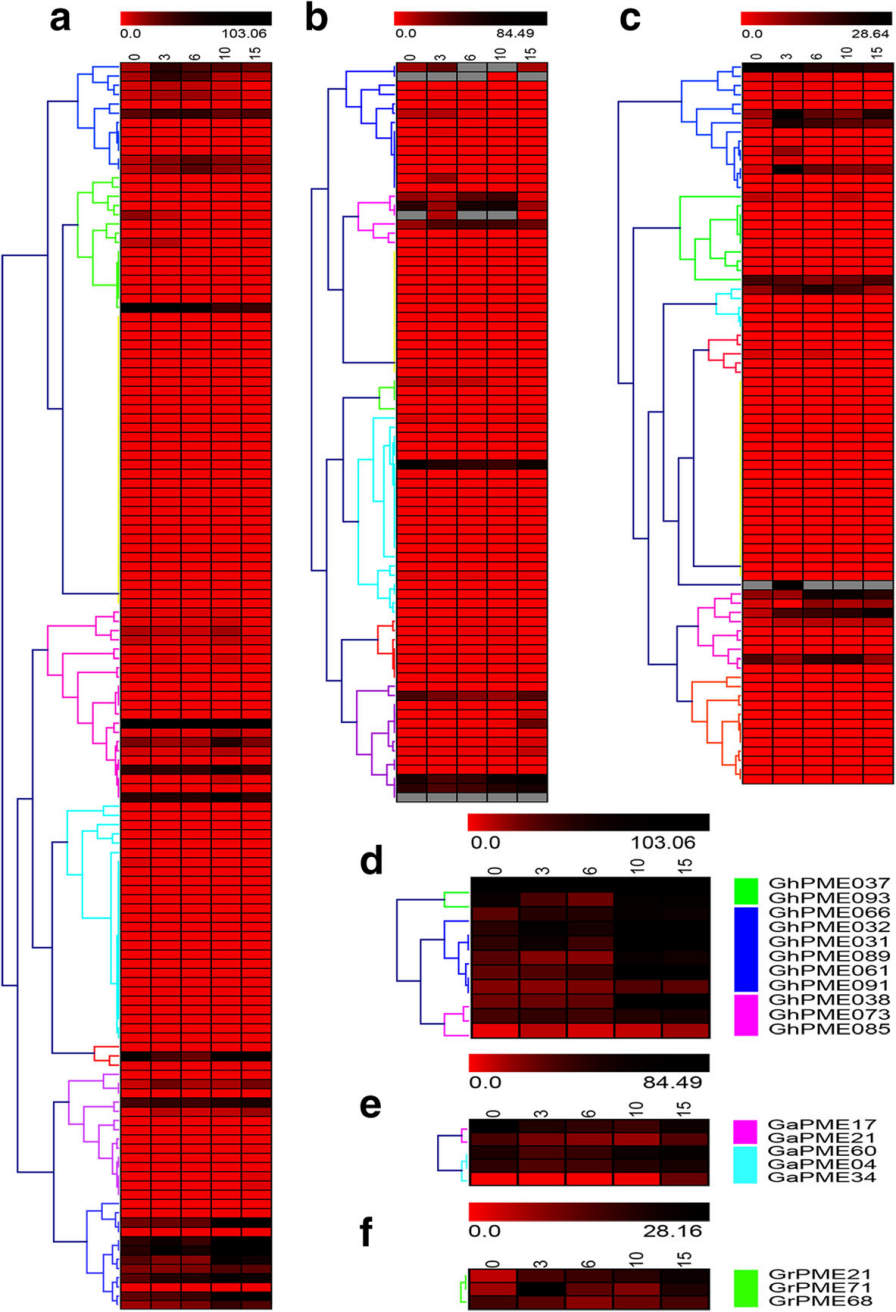
Expression patterns of the *PMEs* family in *G. raimondii*, *G. arboreum* and *G. hirsutum*. **a** Heatmap showing the clustering of 135 *PMEs* of *G. hirsutum* across five tissues (ovules at 0 DPA, 3 DPA, 6 DPA, 10 DPA, and 15 DPA; mentioned at the top of each lane). **b** Heatmap showing the clustering of 80 *PMEs* of *G. arboreum* across five tissues (ovules at 0, 3, 6, 10, and 15 DPA; mentioned at the top of each lane). **c** Heatmap showing the clustering of 78 *PMEs* of *G. raimondii* across five tissues (ovules at 0, 3, 6, 10, and 15 DPA; mentioned at the top of each lane). **d** Expression of 11 (*G. hirsutum*) *PMEs* is predominantly expressed at 15 DPA. **e** Expression of 5 (*G. arboreum*) *PMEs* is predominantly expressed at 15 DPA. **f** Expression of 3 (*G. raimondii) PMEs* is predominantly expressed at 15 DPA. The color scale at the bottom of the dendrogram shows the relative expression levels. RNA-seq data under the series accession number SRA180756 was obtained from the NCBI Sequence Read Archive (SRA) database

All of the identified *PMEs* of *G. hirsutum* were verified by transcriptome data. Expression of 75.56% (102 of 135 genes) in *G. hirsutum* (Fig. 4a) can be detected 0 from 15 day post anthesis (DPA) during fiber development. We detected the expression of 82.5% (66 of 80) *PMEs* of *G. arboreum* (Fig. 4b) and 71.8% (56 of 78) *PMEs* of *G. raimondii* (Fig. 4c) during fiber development (0–15 DPA). We found that 11 *PMEs* of *G. hirsutum* (Fig. 4d) and five *PMEs* of *G. arboreum* were predominantly expressed in fiber development at 15 DPA (belong to the period of secondary wall thickening) (Fig. 4e). However, only three genes in *G. raimondii* showed higher expression at 15 DPA (Fig. 4f).

**Fig. 5:**
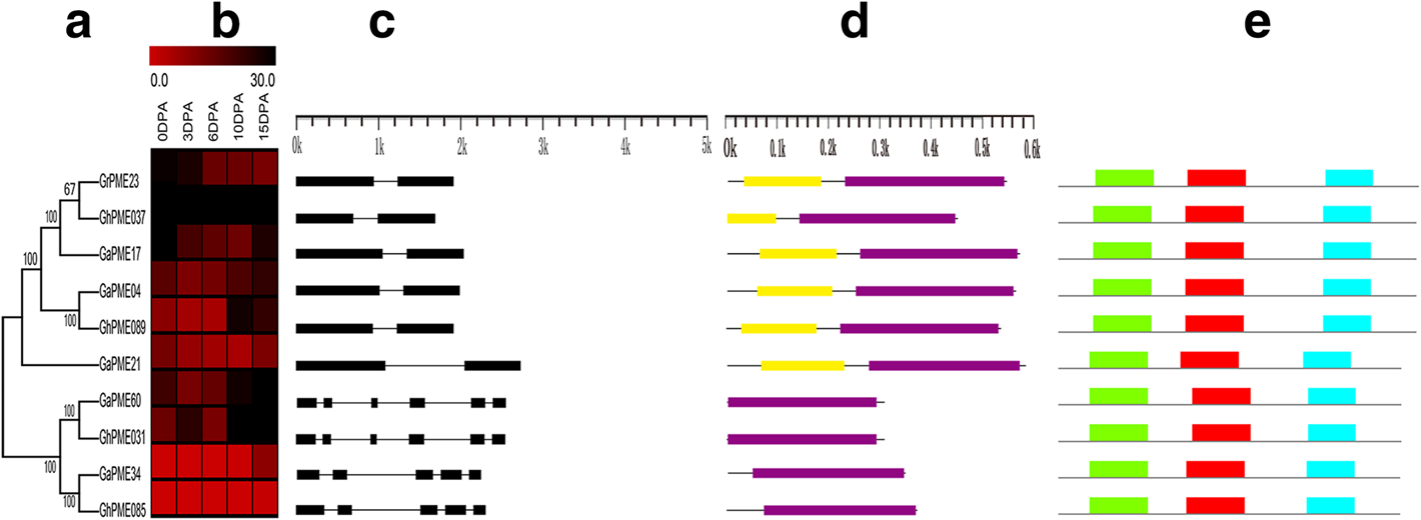
Analysis of *PMEs* predominantly expressed in fiber. **a** The phylogenetic tree was constructed with MEGA 5.1. **b** Heatmap showing the clustering of *PMEs* across five tissues (ovules at 0, 3, 6, 10, and 15 DPA; mentioned at the top of each lane). The color scale at the bottom of the dendrogram shows the relative expression levels. **c** Exon/intron structures of *PMEs* predominantly expressed in fibers. Exons and introns are represented by boxes and black lines, respectively. **d** PME domain of the PMEs protein. **e** Motif of the PME protein

To examine the differential expression of homologous gene pairs among the three cotton species, *PMEs* of *G. arboreum* predominantly expressed in 15 DPA and its homologous genes were selected for phylogenetic analysis (Fig. 5a). Based on above results, four homologous gene pairs were chose for further studies. The data showed that expression patterns of homologous genes pairs were significantly affected. Moreover, the expression levels of almost all genes in the A genome of cotton were higher than the genes in the D genome, and lower than the genes in the AD genome of cotton (Fig. 5b), for example, the expression of *GrPME23* in fiber development (at 15 days) was 12.28,007, whereas the expression of *GaPME17* was 25.40,384. The expression of *GhPME037* in fiber development (15 DPA) was 46.41,517. The expression level of *GaPME34* was higher than the expression of its orthologous genes in the AD genome of cotton.

To survey on mechanism of the differences among orthologous gene pairs, we compared their gene structure (Fig. 5c), protein domain conservation (Fig. 5d), and sequence motifs (Fig. 5e). The results showed that orthologous gene pairs have minimal to negligible effect on the structure of the genes. The length of the first exon affected the structure of these genes. *GaPME17*, *GhPME037*, and *GrPME23*, *GaPME04* and *GhPME089*, *GaPME34* and *GhPME085* were all different in only this one exon. The conserved domain of the protein between the genes did not differ significantly. Protein of orthologous gene pairs varied only on the position of the conserved domain and the length of the non-conserved region (Fig. 5d).

Analysis of putative cis-element motifs of *PMEs* homologous genes pairs of *G. arboreum* and *G. hirsutum* showed significant differences between their promoter regions (Additional file 6: Figure S2). Thus, we speculated that structure variation in promoter region might affect expression levels of homologous gene pairs.

### qRT-PCR analysis for *PMEs* homologous genes pairs

**Fig. 6:**
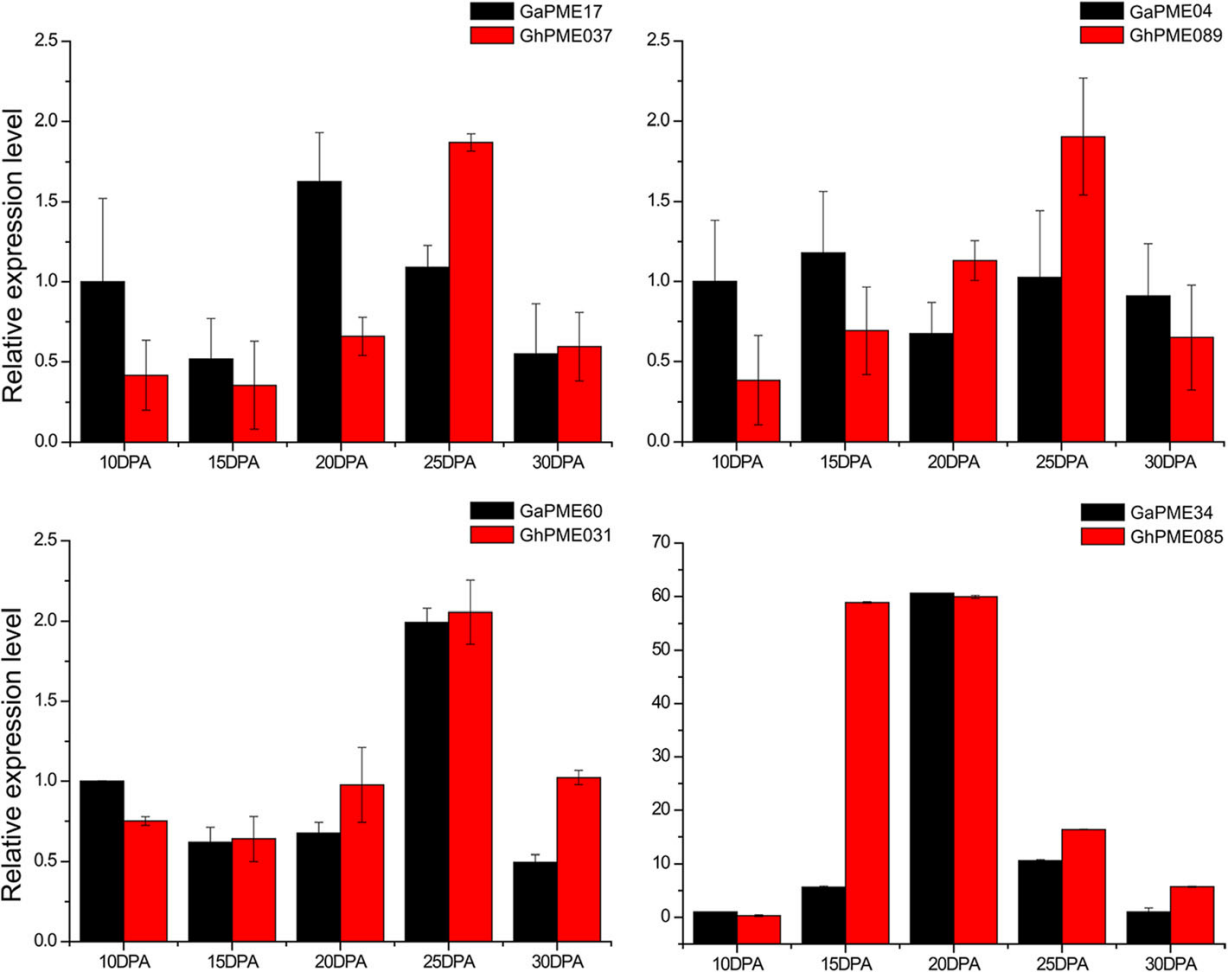
Expression analysis of 4 selected *PMEs* homologous genes pairs using RT-qPCR. The relative mRNA abundance of 4 selected *PMEs* was normalized to the reference gene histone 3 in different tissues. The bars show the standard deviation of three technical replications

To verify the alteration of expression patterns of four *PMEs* homologous gene pairs in *G. hirsutum* and *G. arboreum*, qRT-PCR was employed in this study. The results (Fig. 6) showed that the expression of *PMEs* peaked in Asiatic cotton at 20 DPA and 25 DPA in upland cotton. The average performance of upland cotton was higher than the Asiatic cotton fiber development at 25 DPA suggested that the expression level of Asiatic cotton *PMEs* was decreased in the late development of cotton fiber. However, in upland cotton, the *PMEs* expression level continued to increasing. This probably caused the thickening of fibers in the secondary wall, de-esterification of the pectin in the cell wall, reinforcement of the cotton fiber cell wall; thus, increasing the strength and imparting high quality to the upland cotton fiber.

### PMEs activity

There are differences in PMEs activity in different cotton fiber development periods. Of the increasing in Asiatic cotton fiber development and PMEs activity gradually increased from fiber development at 10 DPA to 25 DPA. However, the PMEs activity decreased at 30 DPA. In upland cotton, PMEs activity continued to increasing (Additional file 7: Figure S3). The reason might be that the Asiatic cotton prematurely ended the secondary wall of the fiber growth causing feedback regulation by the cellulose content and accumulation of pectin.

## Discussion

We used bioinformatics analysis to identify 135 *GhPME* genes from AD genomes, 80 *GaPME* genes from A genomes, and 78 *GrPME* genes from D genomes. Cotton *PMEs* could be divided into four clades in two diploid species and eight groups in the tetraploid species, and all their last subfamilies were restricted to PME without a PMEI domain. We speculated the common hypothesis that PMEs that both PME and PMEI domains appear relatively late in the evolutionary process [23], similar to the species of that observed in *A. thaliana PMEs* [24]. Our analysis showed amount of reduction number of genes (from 81 to 78) as compared with Liu’s study (based on 81 sequences), mainly due to using a more stringent screening criteria. Phylogenetic analysis indicated that these could be divided into four subfamilies (Additional file 8: Figure S4), and the fourth subfamily only contained a PME domain (Additional file 1: Table S1) [1]. Previous studies identified 66, 59, and 89 *PMEs* coding genes in *A. thaliana* [24], *O. sativa* [25], and *P. trichocarpa* [26], respectively. The number of the *PMEs* varied greatly in different species. Previous studies had shown that the whole genome duplication (WGD) and tandem repeats were the main reasons of gene family expansion during the process of plant genome evolution [27, 28]. Plants have a higher rate of gene duplication compared to other eukaryotes [29]. And also, recent studies had shown that genome of diploid cottons had underwent at least two rounds of WGD [18, 19]. The uneven distribution of genes on the chromosome might be due to gene replication or a partial fragment of gene duplication of the cotton genome that occurred during its long evolutionary history. The entire gene sequence of cotton doubled with every copy event and these extra genes were recombined or go undetected over time [21]. The distribution of genes on the chromosome suggested that 19 of 80 *PMEs* appeared as tandem repeats in Asiatic cotton with seven members in Cluster I (*GaPME61- GaPME67*). This was also the main reason for the expansion of this gene family. Arabidopsis *PMEs* family experienced the *α* and *β* replication events [25]. Eight genes were formed by tandem duplication; rice *PMEs* family experienced the events σ and ρ copy events, four gene is made copy tandem formation [25].

The evolutionary tree analysis showed that the *PMEs* sequence within the same species showed high similarity; kinship was also relatively close but was distant from other species. Gene structure analysis showed that most *PMEs* had 2–3 exons, but a few differed in their number of exons, which ranged from 4 to 6. The differences in exon numbers might be due to *PMEs* function and structure as a result of directional evolution. The N-terminus in the evolutionary process was less stringent; some of the family members contain PMEI conserved domains, which might cause changes in the structure and function of *PMEs*. This conservation helps retain the basic functions of the family, enriches the diversity of genes, and reduces the selection pressure.

Comparative genomics had become a highly interesting area in genomics research especially for the study of extensive genome families. Series of important gene families in crops have been studied by comprehensive analysis, for example, *LEA* in soybean [29], *LBD* and *MAPK* in tomato [27, 28], and *MAPKKK* in cotton [26]. Previous studies found that *PMEs* were associated with fiber quality of cotton [1]. Cotton fiber cells are hollow tubular single cells, and their cell wall was the main structure of the mature cotton fiber. Therefore, genes that were directly related to cotton fiber development and regulatory genes, especially the important component of the cell wall-related genes, provided the basis for research and development of cotton fiber and mechanisms affecting its quality. The completion of the cotton genome sequencing enabled the research on PME genes.

PMEs substrate is an important component of the cell wall, which is synthesized in the Golgi complex, and is secreted into the cell wall in the form of methylesters, and quickly de-esterified by PMEs [1]. PMEs plays an important role in regulating plant growth and pectin remodeling [1] Many cell wall-related genes played an important role during cotton cell development [30, 31]. PMEs played a key role in the modification of pectin and formation of the cell wall [16]. Thus, the expression levels of *PMEs* would likely affect the quality of cotton fiber. Two *PMEs*, At2g47550, and At4g02330 were cloned from Arabidopsis. At2g47550 was predominantly expressed in pollen grains, and sometimes in vascular tissues. However, the expression of At4g02330 varied in flower and pod throughout their development in Arabidopsis. At4g02330 was mainly expressed in the flower abscission tissue, stigma, microtubule organization, and pollen grains. At2g47550 might be involved in the development of pollen and pollen tube, while At4g02330 might participate in pectin metabolism of cell walls to achieve the regulation of cell separation and petals falling [32]. The effect of PMEs could be reversed during processing (heating) of fruits and vegetables. Fruits and vegetables require Ca^2+^, which binded to methylester backbone to release carboxyl, and then binds to Ca^2+^ outside the cell to form a calcium bridge between an adjacent pectin chain, thus hardening the cell wall [33]. Liu et al. verified the five *PMEs* by studying the PME enzyme activity at different stages of fiber development, pectin content, and demethylation of pectin in Sea Island cotton and Upland cotton [1]. Their results suggested that these genes might be an important factor governing cotton fiber diameter and length [1]. The high expression of G *raimondii PME4* and *PME5* in fiber development of secondary wall thickening might be related to the cell development [1]. The alignment result showed that all of the cotton *PMEs* shared high similarity to each other. Moreover, structural similarities suggested that other *PME* members in the cotton genome might be associated with the cotton fiber development.

The subfunctionalization of a gene family was prevalent in evolution and gene duplication was the main cause new gene functions [34–36]. Changes in gene expression patterns of the family often occur prior to functional differentiation [34]. This study did not relate to all the features of a *PMEs* family, but only those involved in cotton fiber development during different periods (0–15DPA). We found that 14 (17.5%) genes were not expressed within a certain time frame suggesting gene redundancy of the copy gene. Gene redundancy raised as a result of interference from the external environment and was important for living systems [37]. Three genes were specifically expressed during cotton fiber elongation indicating their primary role in fiber elongation. *PMEs* are up-regulated during fiber development suggesting that pectin formation affects fiber diameter and length [1], and results in longer and thinner fibers. Pectin could be differentially demethylesterified by PMEs to strengthen or loosen plant cell walls [38]. Five genes were highly expressed in fiber development during secondary wall thickening. Secondary wall thickening was a critical period for the formation of fiber strength. High expression of *PMEs* during this period was related to the mechanical strength of the fiber.

Expression patterns of orthologous genes pairs were significantly different between two plant species. The expression level of A genome was higher than the expression level of D genome, the expression level of *G. hirsutum* were higher than the expression of A genome, and only GaPME34 higher than the expression of its orthologous genes. qRT-PCR results (Fig. 6) showed that expression of PMEs genes were difference between diploid and tetraploid cotton. The expression of *PMEs* in Asiatic cotton and upland cotton peaks (20 DPA in *G. arboreum*, 25 DPA in *G. hirsutum*) during fiber development. Of the fiber had a peak, the peak mainly in cotton fiber development 20 DPA. These differences were presumably due to the differences in promoter elements of these genes. The results showed that most *PMEs* are expressed at high levels in secondary wall thickening of the cotton fiber development, perhaps related to the fiber strength during this period.

PMEs decomposed pectin and played an important role in the expansion process in the plant cell wall. *PMEs* had different expression patterns in the process of the cotton fiber formation. However, the specific molecular evolutionary mechanisms and post-transcriptional regulation of gene expression pathways and regulatory pathways of *PMEs* required further investigation.

## Conclusions

This study systematically examined the gene structure, protein domains, physical and chemical properties, gene expression, phylogeny, and collinearity of *PMEs*. The findings provided here will provide an important basis for further research on the function of cotton *PMEs*.

## Methods

### Materials and processing methods

The plant material used in this study was *G. hirsutum* cv 69307 and *G. arboreum* Shixiya I. The material 69307 were from a RIL population developed with the parents 0–153 and sGK9708 and it showed a positive transgressive segregation in fiber strength. The detail information about the population construction was described in Sun’s report [39]. The fiber quality of the parents and the line 69307 was described in Zhang’s report [40]. In the day of flowering, flower buds were tagged as zero DPA. The bolls were collected of each sample (5, 10, 15, 20, 25, 30 DPA) in the morning. The fibers were separated from the ovules, frozen in liquid nitrogen. Before RNA extraction, all the samples were stored at a refrigerator with −80 °C.

### RNA-seq analysis

CTAB method was used to isolated the subsequent total RNA samples from 3 g of cotton fiber (*G. hirsutum TM-1*, *G. arboreum* Shixiya I and *G. raimondii*, 0, 3, 6, 10, and 15 DPA) [41]. A Nucleospin® RNA clean-up kit (MACHEREY-NAGEL, Düren, Germany) was used to purified the total RNA. An Agilent Bio-analyzer (Agilent Technologies, Santa Clara, CA, USA) was used to assess the quality of the RNA sample. After sequencing libraries were prepared following the manufacturer’s standard instructions, and all RNA samples were sequenced on an Illumina HiSeq 2500 platform (Illumina, Inc., San Diego, CA, USA). The CLC Genomics Workbench software 4 (http://www.clcbio.com) was used to analyze the transcriptome data with default parameters. MeV program was used to draw the heat map of the expression data [29].

### RNA isolation and qRT-PCR

The CTAB method was used to extracting the RNA from fiber cell samples [41]. A Nanodrop2000 nucleic acid analyzer was used to test thequality of the RNA sample. A PrimeScript RT reagent kit with a gDNA eraser (TaKaRa, China) was used to performed reverse transcription of samples. The software Premier 5 was used to design primers for the fluorescent quantitative research (Additional file 9: Table S5), and *GhHistone 3* (AF024716) was used as a reference gene. The expression levels of the PMEs were measured by using Applied Biosystems® 7500 Real-Time PCR Systems. Same method was used to analyze expression changes in *G. arboreum*.

### The data related to construct phylogenetic tree

According to evolutionary analysis of *Gossypium*, 11 plants sequenced genome were selected for *PMEs* predicting and further Phylogenetic analysis including *A. thaliana* [42], *O. sativa* [43], *V. vinifera* [44], *P. trichocarpa* [45], *G. max* [46], *T. cacao* [47], *C. papaya* [48], *castor bean* [49] and three cotton (*G. hirsutum* [21], *G. arboreum* [20], and *G. raimondii* [18]). Annotated protein sequences data sets of 11 plant species with sequenced genome were downloaded from corresponding genome database (see Availability of data and materials). And then, all of the protein sequences were used for identification of *PMEs*. Predicted *PMEs* sequences were further phylogenetic tree construction.

### Identification of the *PME* family in cotton

The software HMMER 3.0 was used to predicted proteins which contained PME (PF01095) (http://pfam.xfam.org) and/or PMEI (PF04043) domains with parameter --cut_ga [50]. Only the genes without questionable PFAM annotations (i.e., significant PME and/or PMEI domain but low E-value; low coverage of the domain) were used to do the next analyzes.

### Analysis of *PMEs* family

The data for the D, A, and AD genomes were parsed by a Perl program. The information of *PME*s of the calculation of the chromosome locations and structures was selected. We obtained the homologous genes between different cotton species by blast program (E-value ≤1e-10, Identity ≥ 60%). The collinearity of homologous gene pairs was drawn using CIRCOS package (http://circos.ca). Mapping of *PME* genes was performed using Map Chart [51]. Phylogenetic trees were constructed by employing MEGA software with neighbor joining model, and bootstrap values (1000 replicates) are indicated at each node [52]. Exons and introns were displayed by using The Gene Structure Display Server (GSDS, http://gsds.cbi.pku.edu.cn) [53]. Conserved domains prediction were performed using the SMART Package (http://smart.embl-heidelberg.de) program [54]. Motif analysis was conducted using online tools (maximum number of motifs, three; minimum motif width, six; and maximum motif width, 50) (http://meme.nbcr.net/meme) [55]. The *cis*-acting elements prediction was performed using an online tool PlantCARE (http://bioinformatics.psb.ugent.be/webtools/plantcare/html) [56].

### PMEs activity assay

Based on the Hagerman with some modifications [57], total PMEs enzyme activity was measured. Briefly, 1 g of a fiber sample was taken in a prechilled mortar and 5.0 mL 8.8% (w/v) pre-cooled NaCl was added. The samples were centrifuged for 10 min; the supernatant was collected, and adjusted to pH =7.5 with 0.1 mol/L NaOH to obtain a crude enzyme solution. Four milliliters of 0.5% (w/v) pectin solution and 0.3 mL 0.01% (w/v) bromophenol blue were added to a test-tube, followed by adding 300 uL of an enzyme solution. After 2 min, the absorbance value, and the ΔA620/min∙g expressed enzyme activity, of each sample was analyzed in triplicate [57].

## Additional files

Additional file 1: Table S1.
*PMEs* genes identified from the three cotton species. (XLSX 36 kb)
Additional file 2: Figure S1. Chromosomal location of *PMEs*. a. Chromosomal location of 135 *GhPME* genes. A total of 104 genes are located on normal chromosomes, whereas the other 31 are located on scaffolds. b. Chromosomal location of 80 *GaPME* genes. A total of 79 genes are located on normal chromosomes, whereas the other one is located on scaffolds. (TIF 1445 kb)
Additional file 3: Table S2. Orthologous *PMEs* gene pairs of *G. arboreum* and *G. raimondii*. (XLSX 14 kb)
Additional file 4: Table S3. Orthologous *PMEs* gene pairs of the three cotton species. (XLSX 23 kb)
Additional file 5: Table S4. Selective pressure between *T. cacao* L. and the three cotton species paralogous *PMEs* gene pairs. (XLSX 23 kb)
Additional file 6: Figure S2. Analysis of putative *cis*-element motifs of *PME* homologous genes pairs of *G. arboreum* and *G. hirsutum* promoter. *cis*-element motifs are represented by boxes. (TIF 612 kb)
Additional file 7: Figure S3. Cotton fiber proteins were isolated and used for PME activity assay. Error bars represent the SE of three biological replicates. (TIF 173 kb)
Additional file 8: Figure S4. A phylogenetic tree was constructed with MEGA 5.1 using the neighbor-joining (NJ) method with 1000 bootstrap replicates based on a multiple alignment of 78 amino acid sequences of PMEs from *G. raimondii*. The four major subfamilies are numbered I to IV. (TIF 1228 kb)
Additional file 9: Table S5. Primer pairs used in quantitative real-time PCR analysis. (DOCX 12 kb)

## Abbreviations

AA: Amino acid
DPA: Day post anthesis
*Ga*: *Gossypium arboreum*
*Gb*: *Gossypium barbadense*
*Gh*: *Gossypium hirsutum*
*Gr*: *Gossypium raimondii*
Ka: Nonsynonymous substitution rate
Ks: Synonymous substitution rate
*LEA*: *Late embryogenesis abundant*
*MAPK*: *Mitogen aetivated protein kinase*
*MAPKKK*: *Mitogen activated protein kinase kinase kinase*
PME: Pectin methylesterase
PMEI: Pectin methylesterase inhibitor
qRT-PCR: Quantitative real-time polymerase chain reaction
WGD: Whole genome duplication
