# Supplementary figures and images for: Genome-wide identification, phylogeny, and expression analysis of pectin methylesterases reveal their major role in cotton fiber development
Source: BMC Genomics. 2016 Dec 7;17:1000. doi: 10.1186/s12864-016-3365-z (PMC5142323; doi:10.1186/s12864-016-3365-z)

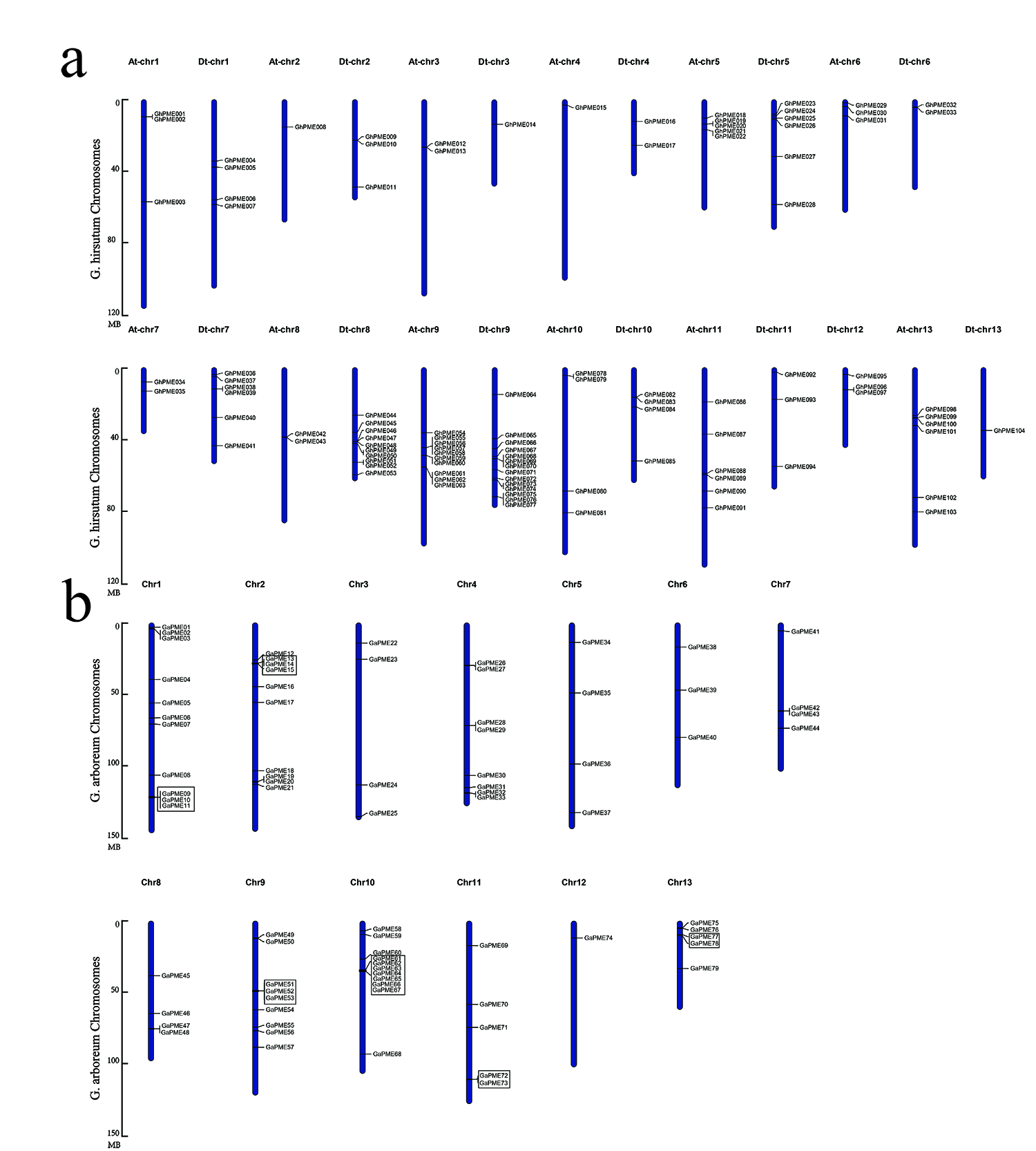

Supplement: Additional file 2: Figure S1. — Chromosomal location of PMEs. a. Chromosomal location of 135 GhPME genes. A total of 104 genes are located on normal chromosomes, whereas the other 31 are located on scaffolds. b. Chromosomal location of 80 GaPME genes. A total of 79 genes are located on normal chromosomes, whereas the other one is located on scaffolds. (TIF 1445 kb) [file 12864_2016_3365_MOESM2_ESM.tif]

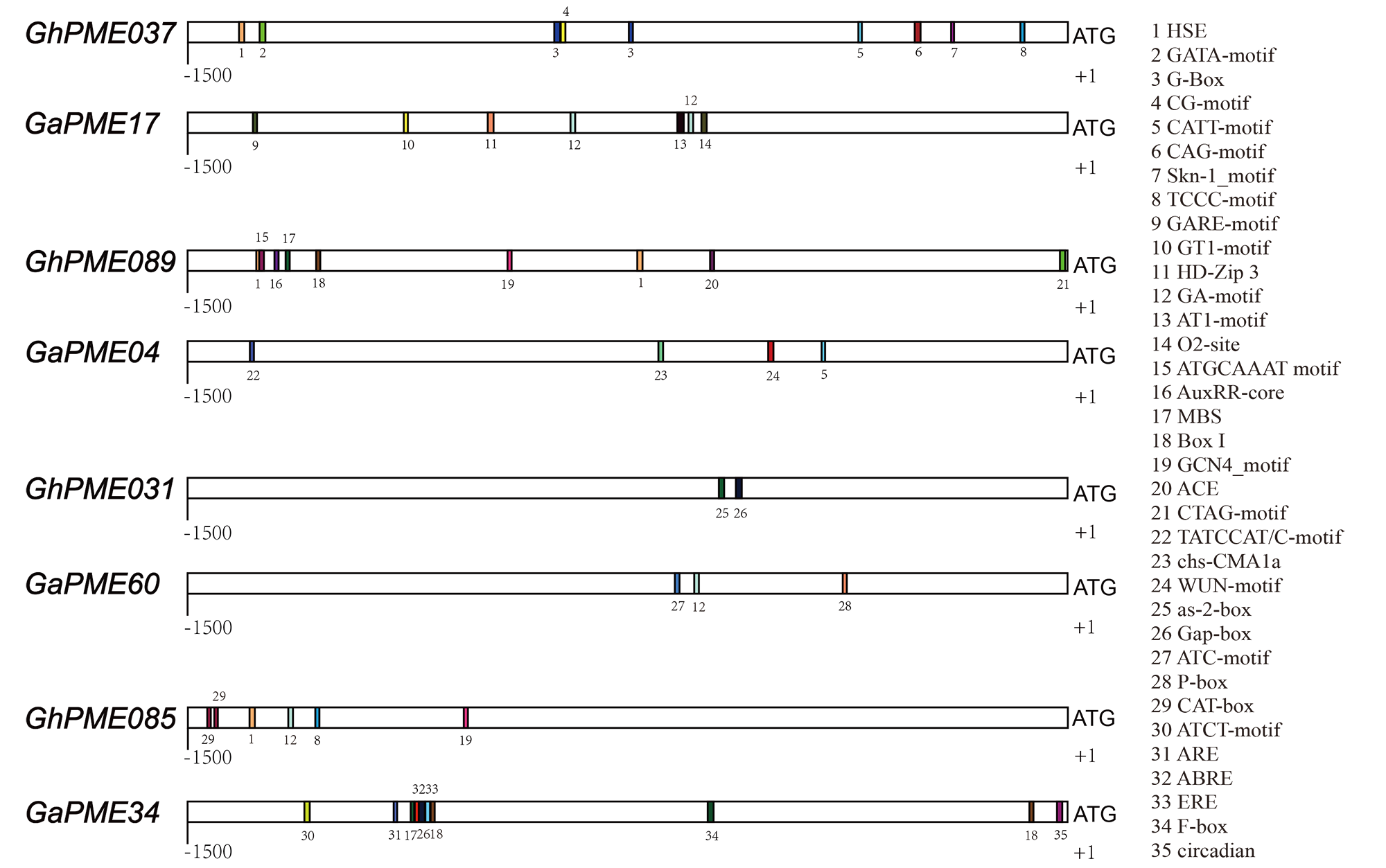

Supplement: Additional file 6: Figure S2. — Analysis of putative cis-element motifs of PME homologous genes pairs of G. arboreum and G. hirsutum promoter. cis-element motifs are represented by boxes. (TIF 612 kb) [file 12864_2016_3365_MOESM6_ESM.tif]

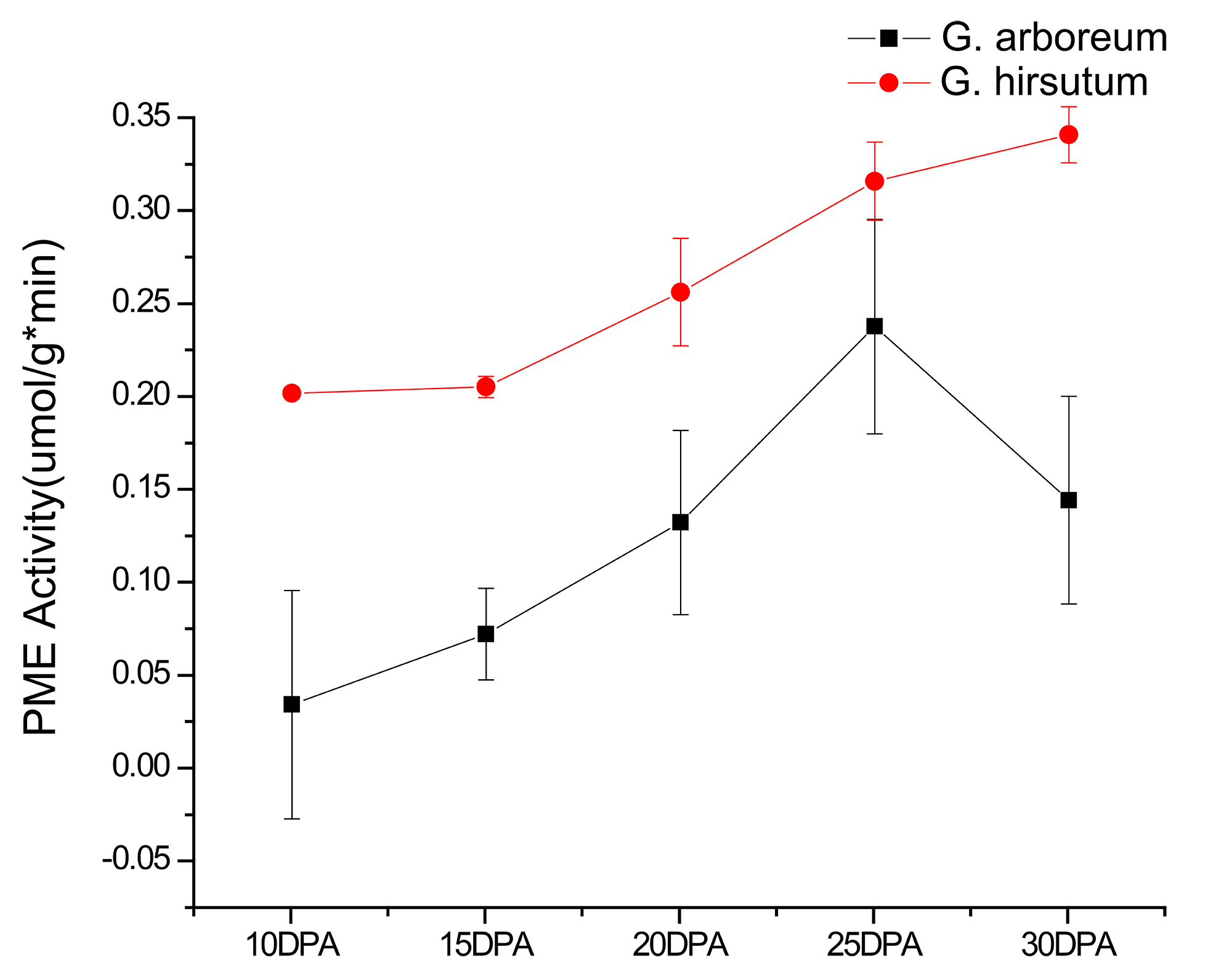

Supplement: Additional file 7: Figure S3. — Cotton fiber proteins were isolated and used for PME activity assay. Error bars represent the SE of three biological replicates. (TIF 173 kb) [file 12864_2016_3365_MOESM7_ESM.tif]

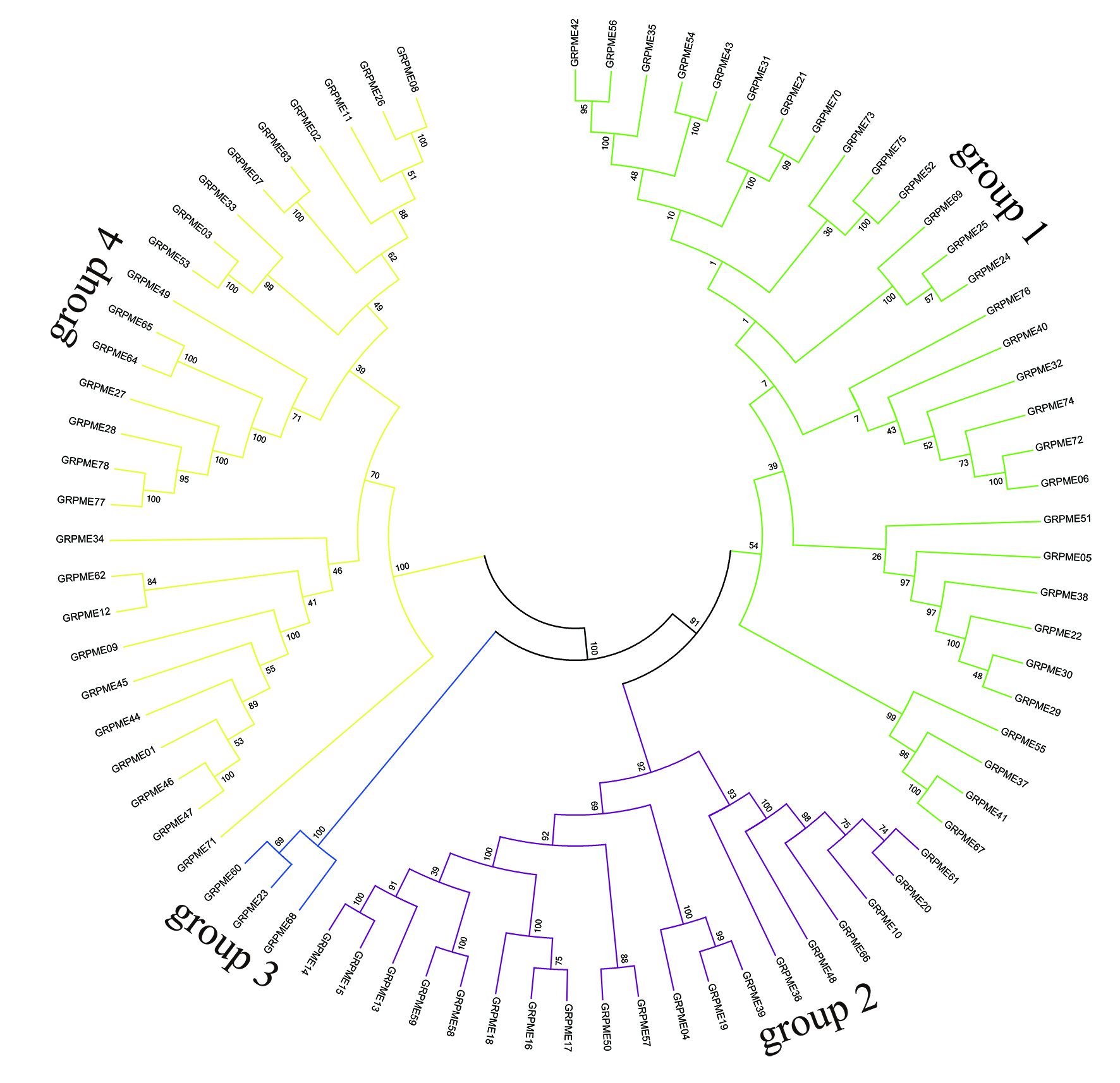

Supplement: Additional file 8: Figure S4. — A phylogenetic tree was constructed with MEGA 5.1 using the neighbor-joining (NJ) method with 1000 bootstrap replicates based on a multiple alignment of 78 amino acid sequences of PMEs from G. raimondii. The four major subfamilies are numbered I to IV. (TIF 1228 kb) [file 12864_2016_3365_MOESM8_ESM.tif]
