# Supplementary material for: Genome-wide identification, phylogeny, and expression analysis of pectin methylesterases reveal their major role in cotton fiber development
Source: BMC Genomics. 2016 Dec 7;17:1000. doi: 10.1186/s12864-016-3365-z (PMC5142323; doi:10.1186/s12864-016-3365-z)
Supplement: Additional file 9: Table S5. — Primer pairs used in quantitative real-time PCR analysis. (DOCX 12 kb) [file 12864_2016_3365_MOESM9_ESM.docx]

Primer pairs used in Quantitative Real-time PCR

| gene | primer | sequence(5'to3') |
| --- | --- | --- |
| GhPME037 | Forward | AGTGCCGTATTCAGGGGAAC |
|  | Reverse | TTTTCGGGTTTTACCTGCCG |
| GaPME17 | Forward | AACTCCCCTGTTTACGCCTC |
|  | Reverse | GGAGGGAGGTCGGATTAGGA |
| GaPME04 | Forward | AGGAAGGGGTGTACGAGGAA |
|  | Reverse | CGCAGACTCGTAAGTGGTCA |
| GhPME089 | Forward | AGGAAGGGGTGTACGAGGAA |
|  | Reverse | CGCAGACTCGTAAGTGGTCA |
| GaPME60 | Forward | GGGGAAAAGCCGAAAACGAG |
|  | Reverse | CCGTTTCGATTGACAGCACC |
| GhPME031 | Forward | GGGGAAAAGCCGAAAACGAG |
|  | Reverse | CCGTTTCGATTGACAGCACC |
| GaPME34 | Forward | AGCCAATCCCTGGTGAAACT |
|  | Reverse | AGAAGTGTGTCTTGGGCTCC |
| GhPME085 | Forward | AGCCAATCCCTGGTGAAACT |
|  | Reverse | AGAAGTGTGTCTTGGGCTCC |
